# Acute Cardiovascular Response to Sign Chi Do Exercise

**DOI:** 10.3390/healthcare3030796

**Published:** 2015-08-28

**Authors:** Carol E. Rogers, John Carlson, Kayla Garver

**Affiliations:** University of Oklahoma Health Sciences Center, 1100 North Stonewall Avenue, Oklahoma, OK 73117, USA; E-Mails: John-carlson@ouhsc.edu (J.C.); Kayla-garver@ouhsc.edu (K.G.)

**Keywords:** exercise, older adults, blood pressure, heart rate, sedentary lifestyle, cross-sectional studies

## Abstract

Safe and gentle exercise may be important for older adults overcoming a sedentary lifestyle. Sign Chi Do (SCD), a novel form of low impact exercise, has shown improved balance and endurance in healthy older adults, and there have been no SCD-related injuries reported. Sedentary older adults are known to have a greater cardiovascular (CV) response to physical activity than those who regularly exercise. However their CV response to SCD is unknown. This study explored the acute CV response of older adults to SCD. Cross-sectional study of 34 sedentary and moderately active adults over age 55 with no previous experience practicing SCD. Participants completed a 10 min session of SCD. CV outcomes of heart rate, blood pressure, rate pressure product were recorded at 0, 5, 10 min of SCD performance, and after 10 min of rest. HR was recorded every minute. There was no difference in CV scores of sedentary and moderately active older adults after a session of SCD-related activity. All CV scores increased at 5 min, were maintained at 10 min, and returned to baseline within 10 min post SCD (*p* < 0.05). SCD may be a safe way to increase participation in regular exercise by sedentary older adults.

## 1. Introduction

Given the aging population in the U.S., it is important to develop interventions to promote healthy aging, including physical activities (PA) that are appropriate for older persons with chronic illness and functional limitations. It is also important to select exercise regimens that are safe and congruent with existing physical limitations, yet effective in bringing about measureable changes in functional outcomes. A preliminary study with sedentary older adults revealed that a novel form of meditative movement exercise, called Sign Chi Do (SCD), improved function and endurance, and increased weekly PA [1].

However, while no studies have reported muscular injuries associated with SCD practice, there are no data on the safety of participation in SCD [1]. Of particular concern, little is known about cardiovascular responses to SCD that could pose a safety risk to older adults. Accordingly, this study evaluated the cardiovascular response (CV) to participating in a single bout of SCD and associated functional fitness outcomes.

### Cardiovascular Response to Exercise

The energy expenditure of exercise requires increased oxygen transportation to meet muscle demands [2]. The CV response to exercise is demonstrated initially by a linear increase in heart rate (HR), blood pressure (BP), pulmonary ventilation (RR), myocardial oxygen consumption (rate pressure product [RPP]), and cardiac output [3], which reaches a steady state within minutes and is maintained until the cessation of exercise. Following the cessation of exercise, the cardiovascular state returns to normal within 10 min, except for BP, which may drop below the basal level for up to two hours [3]. The greater the intensity of the exercise, the greater the energy expenditure and myocardial demands and, therefore, the greater the increase in HR, BP, RR, and RPP.

Aerobic capacity during exercise decreases with age [4], but the rate of decline is less for active persons than for sedentary persons [2,5,6]. Myocardial consumption demands are also higher in sedentary than in physically active older adults. However sedentary older adults, as well as those with cardiac disease, show a higher HR response to exercise due to greater muscle tissue and myocardial demands [3]. Older adults with coronary disease have shown improved exercise capacity following aerobic conditioning [7]. Conditioning results in more efficient oxygen metabolism and myocardial perfusion, which are reflected in a lower CV response [7].

The study reported here measured the CV response to SCD practice among adults age 55 years and older, in order to determine whether the response differed between sedentary and physically active older adults and to examine the association between heart rate (HR) and functional fitness. We expected that adults over 55 would increase their HR, BP and rate RPP in response to a 10 min session of SCD, but sedentary adults over age 55 would show greater increases in HR, BP and RPP than physically active adults, and the HR response would be inversely associated with functional fitness scores.

## 2. Methodology

### 2.1. Design and Sample

This was a descriptive cross sectional study of sedentary and physically active older adults with measurements of CV outcomes before SCD exercise (baseline), and at 5, 10, and 20 min. Associations of the CV outcomes with functional fitness measures were investigated.

The study was approved by the University Institutional Review Board. Findings of this study were previously reported at a conference [8]. Participants were recruited via fliers distributed at a Medical Center and community senior centers. When interested persons called the research office, they were asked pre-screening questions to determine initial eligibility and scheduled for an appointment to confirm eligibility. Individuals meeting inclusion requirements were invited to participate in the study and informed consent obtained.

To be included, participants had to be community dwelling, over 55 years; free from cardiovascular risk (HR between 60 and 100; and normotensive with treated hypertension BP < 180 mmHg systolic and < 90 mmHg diastolic, or untreated hypertension < 160 mmHg systolic and < 100 diastolic); able to walk independently without the aid of a walker or cane; sedentary or participating in low or moderate intensity physical activity; able to safely participate in exercise (as determined by the Physical Activity Readiness Questionnaire [9] and/or clearance to participate from a healthcare provider); and able to speak English. People who reported hard intensity or very hard intensity PA using the Stanford Brief Activity Survey were excluded from the study because they were expected to be too conditioned to respond to low intensity exercise such as SCD. Other exclusion criteria were current or prior history of SCD practice (previous experience with other mind-body practices acceptable); cardiovascular or musculoskeletal disease known to interfere with participation in regular exercise; cardiovascular risk (untreated stage 2 hypertension; pacemaker; any acute cardiac symptoms) or cognitive impairment (scoring less than 3 on a Mini-Cog evaluation for cognitive function [10].

Power analyses indicated that 34 subjects would provide an estimated power of 88% to detect a medium effect size (Cohen’s *f* = 0.25) for the interaction of group and time. Fifty-three adults were screened; 8 were excluded due to current participation in hard to very-hard intensity physical activity (*n* = 6), requiring a cane for mobility (*n* = 1), or cognitive score < 3 (*n* = 1). Of those who met inclusion criteria, 3 were unable to be contacted for scheduling data collection, 3 did not receive clearance for participation from their physician, and 1 did not have transportation to the testing facility. An additional 4 were eligible but were placed on a waitlist since 34 participants were already scheduled for data collection.

#### Categorizing Subjects into Active and Sedentary Groups

The Stanford Brief Activity Survey was used to determine participants’ physical activity level. This survey is designed to assess an individual’s typical daily activity by rating work and leisure physical activity as inactive, light intensity, moderate intensity, hard intensity, or very hard intensity. The Stanford Brief Activity Survey has a reported sensitivity of 0.73 and specificity of 0.61 in identifying intensity level of PA [11]. Participants in the current study were classified as sedentary if they report inactive or light intensity. Physically active participants were those who reported moderate intensity.

### 2.2. Procedures

#### Sign Chi Do Exercise

After signing consent, a HR chest strap and coordinating watch were applied and a BP cuff was applied to the participant’s left upper arm. Participants watched a training DVD to learn the 10-min SCD exercise routine of Healthy-Happy-Holy with transitional leg stances. Once all questions were answered, the baseline BP and HR were recorded and compared to the basal measures to verify that no change had occurred. The participants were then asked to practice the Healthy-Happy-Holy SCD exercise for 10 min while following a recorded DVD to standardize performance. The exercise routine is summarized in Table 1.

**Table 1:** 10-Min Sign Chi Do Exercise Testing.

Warm-up 1.47 min	Front leaning stance	L stance
Begin with Postural alignment. Shoulders relaxed and in alignment with hips and feet and all facing forward. When standing, feet start shoulder width apart. Repeat each 4 times.	Step forward one stride length, toes first and facing forward as knee bends. Maintain feet shoulders width apart. Opposite foot, toes slightly turned out (30°) and leg straight. Shift weight to back foot. Bend back knee and straighten front knee when shifting weight to back foot. Both feet remain flat on floor at all times. Connect breath and arms with leg movements (as weight shifts forward, raise arms and take in a deep breath, as shifting back, exhale).	Step back with one foot, making an L with feet (creating a 90° angle). Feet should be a comfortable distance apart and the heel of the back foot should land in line with front foot. Shift weight to back foot and front foot should be settled on heel with toes pointing up in the air. Most of weight on the back foot with knee bent and front knee is straight. Shift weight from back to front foot and bend front knee as back knee straightens. Connect breath and arms with leg movements (as weight shifts forward, raise arms and take in a deep breath, as shifting back, exhale).
**Sign Gestures 8.13 min**	**Description**	**Integration of Breath**
Healthy, Happy, Holy Practiced from standing position, incorporating the front leaning stance	Healthy: Bring hands to chest with palms open. Extend arms forward in a powerful manner with hands clenched into fist as muscles in forearm and upper arm tighten, assuming the boxers posture. Follow timing of music.	Breath: Breathe in as hands placed on chest. Breathe out as arms extended.
	Happy: Rolling your hands upward and outward, starting low in your abdomen. This rolling motion is continuous.	Breath: Breathe in as hands move in a continuous motion rolling outward. Breathe out with continuous motion.
	Holy: The right index and middle finger (the H sign) glide across the palm of your left hand extending upward and out on an angle.	Breath: Breathe in as the right fingers are placed on the palm of left hand. Breathe out as fingers brush across palm and extend upward and outward.
Check blood pressure and heart rate at 0, 5, and 10 min. (Record heart rate every minute, and up to 10 min following cessation of movement.)		

Note: Adapted from Rogers, Keller, Larkey and Ainsworth, 2012 [1]. A Randomized Controlled Trial to Determine Efficacy of SCD Exercise on Adaptation to Aging.

### 2.3. Measurement

#### 2.3.1. Descriptive Information

Demographic information, health history, and anthropometric measures were obtained at baseline. Demographic information included (1) age in years; (2) gender; (3) education level in years; (4) race; and (5) ethnicity.

#### 2.3.2. Measures

Variables measured included CV response (HR, BP, and RPP) and functional fitness (upper and lower body strength and flexibility, balance, and cardiorespiratory endurance). Perceived exertion was also measured.

#### 2.3.3. Cardiovascular Response

BP was recorded during the SCD exercise test at baseline, 5, and 10 min; and 10 min after the end of the SCD practice. HR was recorded at 1-min intervals during exercise and for an additional 10 min following (or until return to baseline), and following the 6-min walk test. RPP was calculated after the testing procedures. Training HR was calculated at low (40%–54%), moderate (55%–69%), and high (70%–85%) intensity prior to testing procedures for each participant using the Karvonen formula [12].

According to the testing protocol, if a participant exceeded 85% estimated VO_2max_, or BP greater than 180/90, or experienced any chest discomfort, testing procedures were to be terminated. The participant would be asked to walk in place for 2 min or until a reduction in BP and HR was observed. If HR and BP did not return to normal, emergency procedures would be initiated. However, no participants experienced any of these termination criteria.

After the SCD Exercise, participants rested in a seated position for 10 min to allow a return to baseline BP and HR. They were offered snacks and water during this rest period. After the return to baseline BP and HR, participants completed functional fitness performance tests in the following order: lower body strength, upper body strength, lower body flexibility, upper body flexibility, balance, and endurance. The performance measures were demonstrated and participants were asked to do a return demonstration.

The HR monitor chest strap and wrist watch were applied on the left wrist unless the BP was taken on the left wrist. An aneroid sphygmomanometer (American Diagnostic Corporation) BP cuff and stethoscope were used to measure systolic and diastolic BP. For baseline BP, participants were seated quietly in a chair for 20 min before measurements were taken. During the testing procedures, the participant were in the standing position as recommended by the American Heart Association [13]. The RPP was calculated as the product of HR (BPM) and systolic BP (mmHg) at baseline and every time the BP was recorded during and post the 10 min SCD practice. The peak RPP (maximum HR and BP recorded during testing) was calculated.

#### 2.3.4. Perceived Exertion

To determine participants’ perception of exercise intensity throughout the study, the subjective rate of perceived exertion (RPE) scale was used [9]. RPE was recorded at baseline, post warm up, every minute during the 10 min exercise, 10 min post exercise, and following the six minute walk (6-min) test.

#### 2.3.5. Functional Fitness

Functional fitness was assessed using a battery of valid and reliable tests as developed [14]. Upper and lower body strength were assessed by arm curls and chair sit and reach. Flexibility was assessed by back scratch and chair sit and reach. Balance was measures by the 8 foot timed up and go. Endurance was measured with the 6-min walk test.

Participants completed all assessments during one visit, and they received a $100 gift card upon completion of the data collection session to thank them for their time and travel. Data were collected by the first author and RAs trained to adhere to the protocol. Data were collected on two mornings a week (8 am–noon) from June to December 2013. All testing and data collection were done in two large multipurpose rooms at the College of Nursing. The rooms were climate controlled between 68 and 75 degrees and provided space to move freely. Data collection followed prescribed times and scale administration order.

### 2.4. Statistical Analysis

Deviations from baseline of the HR measurements taken every 1 min during SCD were plotted and modeled as a function of time (minute), activity level (sedentary *vs.* active) and the interaction of time and activity level in linear mixed models (LMMs). The plots showed that a large portion of the change in HR during the SCD session occurred between baseline and the first 5-min measurement. A model with HR segmented at 5 min confirmed that comparatively little change occurred after that point. Similar patterns were seen for SBP, RPP and RPE, so for parsimony the primary tests of the outcomes were done by multivariate repeated measures ANOVA in which the baseline and 5-min measurements were modeled as a function of time, activity level and their interaction. To examine the effects of activity level on baseline outcomes, ANOVA was used to compare baseline values of the cardiovascular outcomes between active and sedentary participants. The levels of the outcomes at10 min after the end of the SCD session were compared to baseline levels in multivariate repeated measures ANOVAs. The association of the functional fitness measures with response of HR to SCD was investigated by correlating the functional fitness measures with change in HR from baseline to the 5-min and 10-min HR measurements.

## 3. Results

### 3.1. Sample Characteristics

A total of 34 participants from a large metropolitan health science center (*n* = 22) and community senior centers (*n* = 12) were enrolled in the study. The participants’ ages ranged from 55 to 83 years with an average of 64.03 ± 7.29 years. As indicated in Table 2, the majority were female and Caucasian. The education level of most of the participants was either college (*n* =16) or graduate school (*n* = 11). Over half of the participants reported a history of cardiovascular disease and/or arthritis. Some reported having high BP that was currently being treated with medication (*n* = 15) while a few were taking medication for an irregular HR (*n* = 5). The participants were primarily non-smokers (*n* = 30) and reported occasional (*n* = 16) or no (*n* = 17) alcohol use. Reported activity level was mostly considered moderate or light activity. One person reported occasionally needing a cane or walker, but could walk independently. Baseline BP and HR for the participants were within normal ranges. There were significant differences between activity level groups on prevalence of CV and respiratory disease, and on pain. All of those conditions were more prevalent in the low activity group. However, there was no significant association of CV disease, respiratory disease or pain with baseline levels of the outcome variables. Therefore the analysis did not control for those factors.

**Table 2:** Baseline Sample Characteristics (*N* = 34).

Variable	Sedentary (*n* = 18) M (SD)/Frequency	Active (*n* = 16) M (SD)/Frequency	Total (*n* = 34) M (SD)/Frequency
Age	63.2 (6.2)	65.0 (8.5)	64.0 (7.3)
Gender			
Women	15 (83%)	12 (75%)	27 (79.4%)
Race			
African American	1 (6%)	1 (6%)	2 (5.9%)
American Indian/Alaska Native	3 (17%)	0	3 (8.8%)
Caucasian (White)	14 (78%)	14 (88%)	28 (82.4%)
Other	0	1 (6%)	1 (2.9%)
Ethnicity			
Latino	1 (6%)	0	1 (2.9%)
Not-Latino	17 (94%)	16 (100%)	33 (97.1%)
Years of Education Completed	16.8 ± 3.1	16.7 ± 2.1	16.7 (2.6)
Chronic Disease			
Cardiovascular *	13 (72%)	6 (38%)	19 (55.9%)
Respiratory *	9 (50%)	1 (6%)	10 (29.4%)
Cancer	1 (6%)	2 (13%)	3 (8.8%)
Diabetes	1 (6%)	0	1 (2.9%)
Arthritis	11 (61%)	7 (44%)	18 (52.9%)
Chronic Pain *	7 (39%)	0	7 (20.6%)
BMI *	32.2 (7.2)	26.3 (5.2)	29.4(7.0)
Systolic Blood Pressure	127.2 (11.5)	126.9 (17.1)	126.6 (13.8)
Diastolic Blood Pressure	76.7 (7.1)	74.9 (6.3)	75.5 (6.5)
Heart Rate	72.8 (7.6)	74.2 (8.9)	73.4 (8.1)

Note. Significant difference between groups (*).

### 3.2. Cardiovascular Response

None of the participants reported complaints of shortness of breath or chest pain during the testing procedures. All participants remained at or below 40% of their estimated VO_2max_ and BP 190/90. Therefore, no testing procedures were terminated due to unsafe CV responses. There were no significant differences between groups on baseline scores for the HR, systolic SBP, or RPP (Table 3). Further, there was no significant differences between activity groups in changes in those measures during SCD. Therefore, the findings for changes during SCD are reported for the total sample.

During SCD, mean HR, SBP, and RPP increased during the first 5 min, after which little increase was seen. Heart rates were significantly higher than baseline after 5 min of SCD by 14 beats, F(1,32) = 59.57, *p* < 0.01, but they were within 2 beats of baseline following the 10-min rest period. SBP increased more than 10 mmHg during the first 5 min of SCD, F(1,32) = 12.16, *p* < 0.01, but was 4 mmHg lower than baseline 10 min after the end of SCD. The pattern of RPP during SCD was similar to its components. Baseline scores ranged between 6600 and 11988 and rose significantly during the first 5 min, F(1,32) = 45.6, *p* < 0.01.They were 7308 and 1820 at 10 min however, at 10 min post SCD, RPP was 9.5% lower than baseline, F(1,30) = 5.18, *p* = 0.03.

**Table 3:** Cardiovascular Response Outcomes *N* = 34 M (SD).

Variable	Baseline	5 Min	10 Min	20 Min
HR Active	73.2 (7.2)	88.7 (13.7)	88.2 (16.2) +	71.4 (6.9)
Inactive	73.7 (9.0)	86.3(11.7)	87.3 (12.0)	72.3 (10.3) ++
Total	73.4 (8.1)	87.4 (12.5)	87.7 (13.8) †	71.4 (8.6) †
SBP Active	125.7 (15)	133.6 (21.8)	137.2 (20.9)	123.8 (13.5)
Inactive	127.4 (13)	140.4 (20.7)	138.4 (17.9)	122 (14.2) ++
Total	126.6 (13.8)	137.2 (21.2)	137.9 (19.1)	122.9 (13.7) †
RPP Active	9155.6 (1078.9)	11918.3 (2991.0)	12318 (3044.7) +	8835.1(1261.5)
Inactive	9419.6 (1671.9)	12175.0 (2613.6)	12109 (2383.7)	8810.4 (1525.7) ++
Total	9295.4 (1409.6)	12054 (2757.2)	12201 (2648.5) †	8822.8 (1377.1) †
RPE Active	0.8 (2) +++	3.6 (7) +++	4.5 (7) +	
Inactive	0.7 (2)	3.9 (10)	4.1 (7) ++	
Total	0.7 (2) ††	3.8 (10) ††	4.9 (7) †††	

Note. Active group *n* = 16, inactive group *n* = 18 except + (*n* = 14); ++ (*n* = 16); +++ (*n* = 15); † (*N* = 32); †† (*N* = 33); ††† (*N* = 30). HR = heart rate, SBP = systolic blood pressure, RPP = rate pressure product, RPE = rate of perceived exertion.

Like the physiological responses to SCD, the rate of perceived exertion (RPE) of participants rose rapidly during the first 5 min, from 0.70 to 3.80, and then more slowly thereafter, to 4.28. The increase during SCD was significant: F(1,30) = 105.58, *p* < 0.01. The association of RPE with HR was weak at best; the correlation between the two was positive at each minute of SCD, but no correlation was larger than 0.14 and none were statistically significant. The sedentary group showed a slightly lower HR response at 3–4 min, corresponding to a lower perceived exertion score. There was a 1-point reduction in HR at 5 min, which was recovered at 6 min.

### 3.3. Functional Fitness

There was a significant difference between the groups in 6-min walk scores (Table 4), although none of the other scores differed between the groups. Correlations of measures of functional fitness with heart rate before SCD and the change in HR after 5 and 10 min of SCD are shown in Table 5. Rank order correlations are presented for fitness measures for which extreme cases were observed. No association of functional fitness with baseline HR was found; the correlations were small to moderate and only one of the six reached the nominal probability level. There also was little support for an association of functional fitness and cardiovascular response to SCD in the middle of the session or at the end. The coefficients were small to moderate and none differed from zero at a conventional level of reliability.

**Table 4:** Functional Fitness Descriptive (*N* = 34, Inactive *n* = 18, Active *n* = 16).

Variable	Mean (SD)	Tertile n (%)		
Chair Stand		1	2	3
Inactive	11.0 (2.9)	12 (35%)	6 (18%)	0
Active	13.4(6.2)	8 (23%)	6 (18%)	2 (6%)
Total	12.2 (4.8)	20 (59%)	12 (35%)	2 (6%)
Arm Curls				
Inactive	12.6 (2.8)	14 (43%)	4 (12%)	0
Active	14.4 (3.2)	6 (18%)	8 (23%)	2 (6%)
Total	13.4 (3.1)	20 (59%)	12 (35%)	2 (6%)
Sit and Reach				
Inactive	−1.6 (5.0)	9 (26%)	8 (23%)	1 (3%)
Active	0.3 (3.0)	3 (9%)	12 (35%)	1 (3%)
Total	−0.7 (4.3)	12 (35%)	20 (59%)	2 (6%)
Back Scratch				
Inactive	−2.2 (3.0)	7 (20%)	10 (29%)	1 (3%)
Active	−3.3 (5.5)	6 (18%)	8 (23%)	2 (6%)
Total	−2.8 (4.3)	13 (38%)	18 (52%)	3 (9%)
Timed Up and Go				
Inactive	6.2 (1.3)	9 (26%)	7 (20.5%)	2 (6%)
Active	5.2 (1.8)	3 (9%)	7 (20.5%)	6 (18%)
Total	5.8 (1.6)	12 (35%)	14 (41%)	8 (23%)
Six-min Walk				
Inactive	1320.4 (264.9)	17 (50%)	0	1 (3%)
Active	1572.8 (269.0)	6 (18%)	9 (26%)	1 (3%)
Total	1439.2 (292.2) *	8 (23%)	9 (26%)	2 (6%)

* *p* < 0.05.

**Table 5:** Correlations of functional fitness measures with heart rate at baseline and change after 5 and 10 min of SCD, *N* = 34.

Variable	Heart Rate r (*p*-Value)		
Functional Measure	Baseline	Change at 5 min	Change at 10 min
Chair Stand †	0.23(0.19)	−0.04(0.83)	−0.02(0.92)
Arm Curls ‡	0.35 (0.05)	−0.13 (0.46)	−0.06 (0.73)
Chair Sit and Reach ‡	0.16 (0.36)	0.15 (0.39)	0.15 (0.41)
Back Scratch ‡	−0.10 (0.58)	0.24 (0.17)	0.26 (0.14)
Time Up and Go †	0.15 (0.40)	−0.22 (0.21)	−0.28 (0.12)
Six-min Walk ‡	0.02 (0.93)	0.06 (0.74)	0.06 (0.74)

Notes. Data are r (*p*-value). ‡ Pearson product moment; † Spearman rank order.

## 4. Discussion

### 4.1. Cardiovascular Responses during SCD

As expected, the older adults in this study showed an increase in HR, BP, RPP and RPE within 5 min of SCD practice which was maintained or slightly increased during the following 5 min. All outcomes except RPP returned to baseline following 10 min of rest. SCD was performed at low intensity, and the maximal HR remained below 40% for all participants, which is at the low range of low intensity activity (40%–54%).

Only a few studies have tested the effect of other forms of meditative movement on HR. Healthy younger adults (41 and 33 years, respectively) showed a 17–20 BPM increase in HR following 20- and 60-min sessions of continuous Tai Chi and Yoga practice [15,16]. One study of HR changes during 24 min of continuous Tai Chi among practitioners with at least 1 year of experience showed a change in HR from 70 to 115 BPM for older women and from 72 to 120 BPM for men [17]. Another study that included younger (59.1 years) experienced male Qigong and Tai Chi practitioners found that Qigong practitioners had a HR response similar to that of continuous SCD practice (91 and 87 BPM respectively) [18]. However, Tai Chi practitioners showed an average HR of 129 BPM during practice.

In one study, RPP scores increased from 10080 to 29980 following a Bruce treadmill exercise test in adults aged on average 52.8 years with coronary artery disease [19]. Another study of older women (65 years average age) found that average pre-training RPP scores at rest were below 8000 and they increased to 19,354 during Balke exercise testing [20]. RPP at rest and during exercise decreased following a 16 week exercise intervention. Thus, in the current study the increase in RPP to above 12,000 during SCD represents a much lower cardiovascular demand than the increases seen during standardized treadmill tests.

Mild to moderate intensity exercise such as resistance training results in a lower RPP than maximal treadmill exercise [21]. Huggett, Elliott, Overend, and Vandervoort [22] found a baseline RPP similar to that for SCD (9000) in older adults (74.2 years), which increased to 13,490 during isometric exercise and 12,760 during eccentric exercise. They also found that HR increased by 10 BPM and SBP by approximately 30 points during isometric and eccentric exercises. Vallejo found that BP, HR, and cardiac workload (measured by expired ventilation) were lower during eccentric than concentric resistance exercise [23]. Based on these findings, the low HR, BP, and RPP response to SCD in our study indicates that SCD is as safe for sedentary older adults to participate in as resistance training.

### 4.2. Relationship between Cardiovascular Responses and Physical Activity and Physical Fitness

We expected sedentary participants and those with lower fitness levels to show a greater HR response to SCD. However, there were no differences in HR, BP, RPP, and RPE measures between sedentary and physically active participants. One possible explanation for the similar CV response of the groups is that SCD was performed at similar intensity in both groups as indicated by perceived exertion scores. Also, there was a limited difference in fitness levels between the groups.

While not statistically significant, the outcome that showed the largest correlation between HR and fitness level was the six-min walk test. Even though there was a significant difference between groups on this score, the changes in HR were not correlated with performance levels. As stated above, the rationale may be there is not a difference between groups on changes in HR.

### 4.3. Limitations

There were limitations to this study. The use of a dichotomous measurement of sedentary or physically active may have limited the analysis. Also, the groups may have been too homogeneous in fitness levels to see differences. PA level should be measured on a continuous scale in order to identify differences in CV response. The sample consisted primarily of older, Caucasian, non-Latino women, and thus findings may not be generalizable to populations who have more men and different racial and ethnic compositions. The study did not measure the energy expenditure of SCD in novice practitioners although this would help to determine intensity levels. Finally, self-selection bias may have existed since participants were volunteers.

## 5. Conclusions and Clinical Implication

Despite its limitations, the study has shown that SCD, a low impact exercise for novice performers, does not place high demands upon the cardiovascular system, and is therefore is a safe exercise intervention for healthy sedentary older adults. Previous studies have shown improvements in balance and cardiorespiratory endurance [1], and thus this may be an effective intervention for older adults to transition from a sedentary lifestyle to participating in recommended minimum exercise requirements. Further research is needed to measure the cardiovascular response to SCD in pre-frail and frail older adults; and the long term effect of SCD practice on CV response and energy expenditure in all populations.

Funding: This work was supported by a University of Oklahoma Health Sciences Center Seed grant and grant from the Donald W. Reynolds Foundation.
